# On the Measurement of Movement Difficulty in the Standard Approach to Fitts' Law

**DOI:** 10.1371/journal.pone.0024389

**Published:** 2011-10-28

**Authors:** Yves Guiard, Halla B. Olafsdottir

**Affiliations:** Laboratoire de traitement et de communication de l'information, CNRS, Telecom ParisTech, Paris, France; The University of Western Ontario, Canada

## Abstract

Fitts' law is an empirical rule of thumb which predicts the time it takes people, under time pressure, to reach with some pointer a target of width *W* located at a distance *D*. It has been traditionally assumed that the predictor of movement time must be some mathematical transform of the quotient of *D*/*W*, called the index of difficulty (*ID*) of the movement task. We ask about the scale of measurement involved in this independent variable. We show that because there is no such thing as a zero-difficulty movement, the *ID*s of the literature run on non-ratio scales of measurement. One notable consequence is that, contrary to a widespread belief, the value of the *y*-intercept of Fitts' law is uninterpretable. To improve the traditional Fitts paradigm, we suggest grounding difficulty on relative target tolerance *W*/*D*, which has a physical zero, unlike relative target distance *D*/*W*. If no one can explain what is meant by a zero-difficulty movement task, everyone can understand what is meant by a target layout whose relative tolerance *W*/*D* is zero, and hence whose relative intolerance 1–*W*/*D* is 1 or 100%. We use the data of Fitts' famous tapping experiment to illustrate these points. Beyond the scale of measurement issue, there is reason to doubt that task difficulty is the right object to try to measure in basic research on Fitts' law, target layout manipulations having never provided users of the traditional Fitts paradigm with satisfactory control over the variations of the speed and accuracy of movements. We advocate the trade-off paradigm, a recently proposed alternative, which is immune to this criticism.

## 1. Introduction: Fitts' law and the Difficulty of Simple Aimed Movement

Fitts' law is a well-known rule of thumb of experimental psychology discovered by Fitts [1] half a century ago. Celebrated as a remarkably robust empirical regularity, the law states the time it takes people, under time pressure, to reach with some pointer a target of width *W* located at a distance *D*. The duration of aimed movement, Fitts' law says, is linearly dependent on the difficulty of the required movement, quantified by an *index of difficulty* (*ID*):
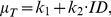
(1)where μ_T_ denotes mean movement time, *k*_1_ and *k*_2_ standing for adjustable constants (*k*_2_>0). The *ID*, which has received various definitions in the literature, is always assumed to be dependent on the ratio of target distance *D* and target width *W*:
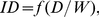
(2)where *f* denotes a strictly increasing function.

Since Fitts [1] there has been agreement in the literature that Fitts' law is of the general form shown in Eqs. 1–2. Note that there is more to these formulas than just a writing convention. The established norm for the formulation of Fitts' law reflects an established norm for the experimental approach to the subject. In what we will call the *Fitts paradigm*, experimenters measure movement time, a random dependent variable, while systematically varying the target layout by manipulating target distance *D* and target tolerance *W*. In other words, the index of difficulty over which experimenters have control in the laboratory (Eq. 2) is assumed to *determine* movement time (Eq. 1).

Thus in the classic Fitts paradigm causality is assumed to flow from right to left across the equal signs of Eqs. 1 and 2. To make this quite explicit, we might have written the above equations as
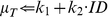
(1′)

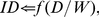
(2′)using the symbol 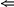 to denote an asymmetrical causal relation.

The present paper focuses on the Fitts paradigm. However, it will be recalled below that other approaches are possible. We will mention two alternative paradigms. One is the well-known Schmidt paradigm [2], which groups the variables of Eqs. 1 and 2 differently, yielding an asymmetrical (causal) dependency of variable error upon the average speed of the movement. The other is the trade-off paradigm recently explored by Guiard, Olafsdottir, and Perrault [3], which construes Fitts' law as a symmetrical (mutual) trade-off between two random variables.

Much of Fitts' law literature rests on the so-called reciprocal task protocol that Fitts himself introduced in his seminal 1954 paper. In the reciprocal (or serial) protocol the participant's task is to alternatively reach two targets, trying to make as many hits as possible in a given lapse of time. But an alternative option is the *discrete*-movement protocol introduced by Fitts and Peterson [4], in which the participant are to make single-shot movements. The discrete protocol is conceptually simplest and, as noticed by Fitts and Peterson [4], it allows more rigorous control over the variables of interest than is possible with the reciprocal protocol. Whereas in the reciprocal protocol μ_T_ is the time it takes not only to carry out a movement, but also to evaluate the error inherited from the previous movement and to prepare the next, in the discrete protocol μ_T_ measures the duration of a pure movement-execution process. Furthermore the meaning of the movement's endpoint spread is interpretable more safely in the discrete case, that variability being generated just by the execution of the movement, whereas in the reciprocal case the spread also reflects, to some unknown extent, the variability of the start point [4], [5]. Below it is the discrete protocol, more suitable for basic research investigations, that will be considered by default.

The subject of the present paper is the measurement of the difficulty of aimed movement within the framework of the Fitts paradigm. This is a theoretical subject in the sense that it requires the discussion of abstract concepts. However, we wish to make it explicit from the outset that we will not depart from an agnostic stance with regard to the explanation of Fitts' law. Why Eq. 1 generally holds—a question for the substantive (causal, compositional) theory, to use Meehl' [6] terms—has been a permanent concern in Fitts' law research, and many proposals have been published [1], [2], [7], [8], [9], [10], [11], [12]. Had the substantive theory been the subject of the present paper, we would have discussed the bridges that link the quantities of Eqs. 1–2 to theoretical entities. But our main subject is measurement, and so we will look in the opposite direction. We will ask instead how these quantities, identified as numbers, map downwards onto the physical, real-world quantities that experimenters concretely manipulate and record in the laboratory, rather than upwards onto theoretical entities.

Consider the fractional expression *D*/*W* of Eq. 2. This mathematical expression stands simultaneously for two things that empirical scientists, as distinct from pure mathematicians, need to carefully distinguish—an abstract number and a concrete physical variable. For a Fitts' law experimenter, *D*/*W* does indeed denote a number that varies from zero to infinity, but it *also* denotes a variable to be manipulated in the laboratory. The problem, as we will see in Section 4, is that the manipulation in question is undoable in practice outside of a rather narrow range: *D*/*W*<3 or so is impossible due to the saturation of movement speed, while *D*/*W*>50 or so is impossible due to the saturation of movement accuracy. Thus, the constraints that affect *D*/*W* qua a number and *D*/*W* qua a physical variable are quite different, justifying the numerical/physical distinction crucial to the next section.

## 2. Scales of Measurement: The True-Zero Issue

Using S.S. Stevens' [13] words, measurement is the process of assigning numerals to objects or events according to certain rules. At issue in this article is the correspondence between the *ID*s of Fitts' law, which are numerical quantities, and the concrete operational quantities they refer to.

A quick reminder of the main four levels of measurement distinguished by Stevens' classic theory of scale of measurement [13] may be useful.

1. The lowest level, designated as *nominal* (or categorical), corresponds to the mere classification of objects that can be sorted but not ranked. For example, in his 1954 study Fitts used three different tasks. Task was a nominal variable, whose modalities were stylus tapping, disc transfer, and pin transfer.
2. Then comes the *ordinal* level of measurement (e.g., cool, warm, and hot) where the variable has levels that obey a transitive-asymmetry rule (if warm>cool and hot>warm, then hot>cool), so that there is only one correct order. Notice that up to this level inclusively nothing is being said about the *spacing* of the various modalities or levels of the variable.
3. The third level of measurement is that using an *equal-interval* scale. One example is temperature on the C° scale, where the difference between 1° and 2° is the same as between 2° and 3°, 11° and 12°, etc. One has a unit and hence a metric, but where the origin or zero of this metric falls is an arbitrary convention (ice melting).
4. The highest level of measurement is that involving a *ratio* scale. That most-severely constrained kind of measurement enjoys all the properties of the first three (i.e., its levels are sorted, ranked, and equally spaced) but in addition it has the special property of a *non-arbitrary zero*. The classic example is temperature as measured on the absolute or Kelvin scale. Not only does the Kelvin scale involve equal intervals, but its zero corresponds to a physical stop—disappearance of vibratory motion at the atomic level. More familiar examples are time duration and spatial distance, of central relevance here.

With regard to scale of measurement, the dependent variable of Fitts' law, movement time, is not an issue. There is little risk saying that mean movement time μ_T_ has a true zero and runs on a ratio scale of measurement. In contrast, it has been unclear so far whether or not this is also true of the quotient of *D/W*, the basic predictor of movement time in the Fitts paradigm.

## 3. Quantifying Task Difficulty in Fitts' law Equations

### 3.1. Mathematical Models

A number of different definitions of the index of difficulty (*ID*) have been proposed in the literature (for example, Plamondon & Alimi [9] list a dozen formulas). Here are four well-known instances:
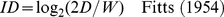
(3)
[1]

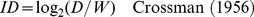
(4)
[14]

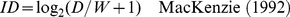
(5)
[15]

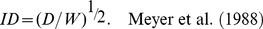
(6)
[10]

Fitts [1] was the first to offer a clear quantitative index of the difficulty of aimed movements. In his seminal 1954 paper, he argued from information-theoretic considerations that the *ID* should be computed as log_2_(2*D*/*W*). Since 1954 most psychologists have been using Fitts' definition of difficulty in Fitts' law experiments [16], even though they have forgotten the binary digit unit of his *ID* [17]. The Crossman variant shown in Eq. 4 was derived from a control theory analysis [7], [18]. MacKenzie [15] offered *ID* = log_2_ (*D*/*W*+1) as an improvement over Fitts' derivation of the *ID* from Theorem 17 of Shannon [19]. Currently MacKenzie's *ID*, known as the Shannon *ID*, is widely accepted in the field of human-computer interaction (HCI), where most research on Fitts' law happens to have taken place over the last three decades [20]. The *ID* of Eq. 6 was derived mathematically by Meyer et al. [10] from an explanatory theory of Fitts' law that has been remarkably influential, the stochastic optimized sub-movement model.

The above four equations define the *ID* as a strictly increasing function of *D*/*W* (Eq. 2). Also invariant among these and other Fitts' law equations is the assumption that movement time must vary linearly with the *ID* (Eq. 1). Combining Eqs. 1 and 2, we may express the law as
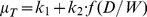
(7)with *k*_2_>0. But the composition of two strictly increasing functions yields a strictly increasing function, and so Fitts' law is a relation of the form
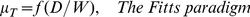
(8)where *f* denotes a strictly increasing function.

### 3.2. Experimental Paradigms

Eq. 8 makes it clear that Fitts' law involves three basic ingredients, a time measure μ_T_ and two length measures *D* and *W*. The dependent variable μ_T_ is the mean of a random variable while on the right-hand side of the equal sign *D* and *W* are determinist quantities assumed to be manipulated by experimenters. But this is one of several possibilities. Eq. 8 characterizes the most popular paradigm inherited from Fitts, but as already noted we also have the Schmidt paradigm [2], where μ_T_ and *W* swap their roles. Movement time becomes a quasi-deterministic, experimentally manipulated quantity while *W*, called “effective tolerance” by Schmidt et al., is the standard deviation of a random variable, movement amplitude. Using the same notation rule as above, the Schmidt paradigm Fitts' law is a relation of the form
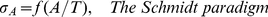
(9)where σ_A_ denotes variable error, or the standard deviation of movement amplitude, while *A* and *T* denote the experimentally-controlled spatial and temporal extents of the movement, both quasi-deterministic variables. Here again *f* stands for a strictly increasing function.

(In the discrete protocol, which uses a fixed identified start point *x*_0_, movement amplitude *A* = *x*_f_−x_0_ and movement endpoint error *E* = *x*_f_−*D* share the same standard deviation σ_A_ = σ_E_, meaning that either is a possible definition of variable error (*VE*). We prefer the former definition *VE* = σ_A_, with which relative variable error takes the form of a regular coefficient of variation σ_A_/μ_A_. Note that in the reciprocal protocol the equality σ_A_ = σ_E_ is not true.)

Another variation is the *trade-off paradigm* recently proposed by Guiard et al. [3], which involves two random variables, one on each side of the equal sign:
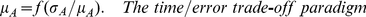
(10)


Eq. 10 assumes a symmetrical relation between two stochastic quantities. Unlike Eqs. 8 and 9, this equation does not exhibit a dependent variable on one side and a predictor on the other. Rather, its two sides are assumed to trade for each other and hence to predict each other, a relation which we might have noted *μ_T_* 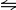
(*σ_A_*/*μ_A_*).

Eqs. 8–10 identify three distinct *paradigms* of Fitts' law research, not just three ways of formulating Fitts' law, but really three different experimental approaches to the problem of simple aimed movement. We see now that the task difficulty issue concerns not Fitts' law in general, but quite specifically the Fitts paradigm summarized by Eq. 8. The task difficulty concept is involved in neither the Schmidt paradigm, which considers variable error and movement speed, nor the time/error trade-off paradigm, which considers movement time and relative variable error.

In fact, awareness that the problem of simple aimed movement can be tackled with the alternative approaches of Eqs. 9 and 10 helps realize that there is something subtly misleading in the familiar assumption that the *ID* captures task difficulty. There is no question that a lower *ID* demands less accuracy. This, however, does not mean that the required movement should be less difficult. Fitts task instructions asking participants to move as fast as they can, given the tolerance, the net difficulty of the movement task must be assumed to be constant across all *ID* values. Thus what the traditional Fitts paradigm calls “task difficulty” only takes account of the accuracy component of the movement task. It is useful to bear in mind that a lower-*ID* task is in fact no less ‘difficult’—it just requires of participants a different balance of effort between speed and accuracy.

### 3.3. A Single Independent Variable in Fitts' Law

Every known variation of Fitts' law, including those delivered by the non-standard paradigms of Eqs. 9 and 10, involves the three basic measures singled out in Eqs. 8–10, namely, a time measure (*T* or μ_T_) and two length measures relating to movement amplitude (*D* or μ_A_) and movement endpoint variability (*W* or σ_A_). At this point one should realize that whenever a fractional expression like *D/W*, *A*/*T*, or σ_A_/μ_A_ appears in a Fitts' law formula, one faces uncertainty as to the *number of physical variables* that the fractional expression is supposed to stand for, as recently emphasized by Guiard [21].

Fitts' law students, who are empirical scientists rather than pure mathematicians, need to care about how the abstract symbols of their models relate to the concrete variables they measure and manipulate in the laboratory. Taking the example of Eq. 8, of special interest in this article, they should be concerned that the conventional mathematical notation *D*/*W* is ambiguous. *D*/*W* may be taken to denote two numbers, the operands of a (doable) division. In this reading of the equation, of the form μ_T_ = *f* (*D*,*W*), two independent variables offer themselves for manipulation in the laboratory, target distance and target tolerance, each of which has the physical dimension of length. But alternatively *D*/*W* may be taken to denote a single number, the quotient of the (done) division. The equation being now of the form μ_T_ = *f*(*Q_D_*_/*W*_), where *Q* denotes a quotient, a single number is left on the right-hand side of the equation. This is an invitation to manipulate a single independent variable, namely relative target distance, which is physically dimensionless. The two options are equally sensible, but they logically cannot be hybridized [21].

Failure to recognize this has led to logical dead ends. For example Meyer et al. [10] used the classic paradigm to try to evaluate experimentally not only the effect of the *ID*, dependent on the quotient of *D/W*, but also the effects of both the numerator *D* and the denominator *W*. The problem with this analysis is that it involved one too much experimental factor, as only two variables can be independently manipulated on the right-hand side of Eq. 8: either the quotient of *D*/*W* and scale (e.g., *D* but not *W*, or *W* but not *D*) or, in an alternative approach, target distance *D* and target width *W*, in which case one must forget about the quotient [21].

Since so far most authors of the literature have assumed Fitts' law to be the dependency of μ_T_ upon the quotient *Q_D_*_/*W*_, a correct tacit assumption [21], from now on this paper will assume that the expression *D*/*W* of Eq. 8 does indeed stand for a single number and refers to a single physical quantity. In the Fitts paradigm of Eq. 8, the experimental variable expressed by the quotient of *D/W* is *relative target distance*, that is, target distance expressed in units of—or scaled to—target tolerance. In the Schmidt paradigm of Eq. 9, the quotient of *A*/*T* measures the *average speed* of the movement. In the tradeoff paradigm of Eq. 10, the quotient of σ_A_/μ_A_ measures the movement's *relative variable error*. The equations being recognized to display a single number on their right-hand side, we may say that, in general, Fitts' law is a relation of the form
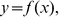
(11)where *f* denotes some strictly increasing function representing either an asymmetric determination relation as in Eqs. 8 and 9, or a mutual influence relation as in Eq. 10.

The merit of Eq. 11, which flatly ignores all the specifics of Fitts' law, is to make it explicit that Fitts' law equations, no matter the paradigm, involve no more than *one* physical measure on each side of the equal sign. This is the case of Eq. 8, which characterizes the Fitts paradigm on which we focus below. The issue being the metric of difficulty, we will ask how one number, the quotient of *D/W*, maps onto a certain dimensionless physical quantity, which we call relative target distance.

## 4. Manipulating Task Difficulty in the Real World

Our purpose in this section is to show that the range over which the *ID* can be manipulated in actual practice by experimenters is very narrow, and to explain why.

### 4.1. Geometrical Limits of Target-Tolerance Manipulation

An *ID* is not just a number, it is a *measure* in the sense that it involves both a numerical and a physical continuum. A certain mathematically transformed quotient, the *ID*, serves to quantify a certain relational property of the target layout.

The ingredients from which *ID*s are computed are two simple collinear lengths, *D* and *W*. Compare the difficulty of a Fitts task with the temperature of a piece of matter, a classic example of metrology. Temperature is both a numerical quantity and a physical quantity and to ask about the metric of temperature is to ask how the former maps onto the latter, but notice that it is rather hard to represent rigorously the physical facet of temperature. Inquiring into the metric of task difficulty is easier because the correspondence one must examine is between a numerical continuum and a geometrical continuum easy to represent graphically.

**Figure 1:**
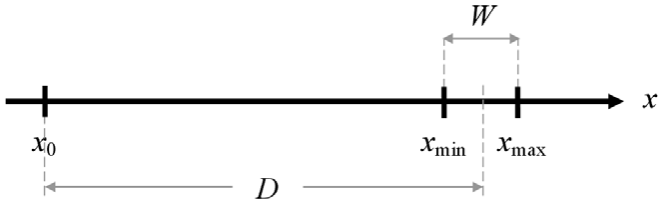
The two basic collinear lengths of the Fitts paradigm.

To begin with, consider the permitted ranges of variation of the two basic lengths *D* and *W*. The very simple aiming task which has served since Fitts to establish Fitts' law is strictly one-dimensional, the dimension being typically spatial. (The continuum need not be spatial, however, as noted by Fitts [1] (his Footnote 4); for example, Fitts' law is known to hold with isometric force [20], and it could be studied along the continuum of musical pitch [21].) The task is to move some pointer (e.g., a screen cursor) in as little time as possible from a fixed start point *x*_0_ to a specified target interval delimited by a minimum and a maximum, as shown in Figure 1. The pointer must reach a final position *x*_f_ such that *x*_min_≤*x*_f_≤*x*_max_. On the continuum the three points *x*_0_, *x*_min_, and *x*_max_ determine two lengths, which the literature conventionally defines as the distance *D* from the start point to target center and target width *W*. Task difficulty is manipulated by varying the arrangement of the three points along the continuum. It is intuitively obvious that, all other things being constant, aiming difficulty will increase as *D* is increased and/or *W* decreased.

**Figure 2 pone-0024389-g002:**
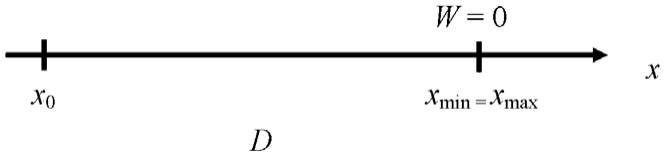
The true zero of tolerance.

Let us ask about the boundaries of the manipulation of *D* and *W*. Figure 2 shows that, no matter the value of *D*, experimenters may reduce *W* as much as they like, down to *W* = 0, where *x*_max_ and *x*_min_ merge. Although the zero-tolerance case cannot be realized *exactly* in the laboratory, it can be very nearly approached, as was the case for example in the Schmidt et al. study [2]. Suppose that on a screen display the three points are marked along the *x* axis with three 1-pixel thick vertical lines. The zero-tolerance condition will obtain when the *x*_min_ and the *x*_max_ lines appear at the same abscissa. In such a case the tolerance *W* = *x*_max_−*x*_min_ will be exactly zero pixel, although the real tolerance will be in fact slightly above zero, actually equal to pixel size. Thus it is fair to say that target tolerance in the Fitts paradigm has a true zero—i.e., a physical zero that is both well defined conceptually and approachable in practice.

**Figure 3:**
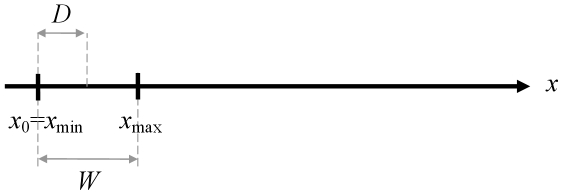
The geometrical minimum of target distance *D* = ½*W*.

In contrast, experimenters cannot reduce *D* down to zero. Since in the paradigm target distance serves to specify the desired amplitude of the movement, the case *D* = 0 makes no sense in principle, simply because a zero-amplitude movement is not a movement. In fact, as visible in Figure 3, any aimed-movement task with *D*≤½*W* is problematic because the task requirement (to reiterate, that *x*_f_ be such that *x*_min_≤*x*_f_≤*x*_max_) would be satisfied from the outset, allowing participants to legitimately ask why they should move at all.

The above definition of an aiming task might be judged incomplete. Experimenters often add to their task instructions the special recommendation to aim to target center (to our knowledge this procedural detail is quite common in Fitts' law experimentation, but it is an informal detail that most authors omit to mention in their reports). This means that a target layout with *D*<*W*/2 might possibly make sense, geometrically. But this is of little importance because, for reasons which we will see in the next section, that case is of no utility whatsoever in practice. It may be firmly concluded that target distance *D*, unlike target tolerance *W*, *cannot* be cancelled out in the Fitts paradigm of Eq. 8.

Since in Fitts' law the predictor of μ_T_ is a dimensionless quotient, it is useful to reformulate the above in terms of relative quantities. For the discrete movement protocol, the practicable range of relative target distance *D*/*W* is ]**½**;+∞[ and the range of relative target tolerance *W*/*D* is ]0;2[. We will see in Section 5.1 that with the reciprocal protocol the range of *W*/*D* is even smaller, being halved.

Any relative layout of the three points that serve to specify a Fitts task is uniquely specified, independently of scale, by either *D/W* or *W/D*. Note that from now on we will use by default the latter description, if only because the range is more convenient.

### 4.2. Human Performance Constraints

Thus far we have considered the range of difficulty that is geometrically available in the Fitts paradigm. But the performance limitations of people further impose their tough constraints on experimenters, who in practice investigate only a small subset of this range.

Table 1 shows the minima and the maxima of relative target tolerance *W/D* reported in a sample of Fitts' law studies, separating the discrete and the reciprocal protocol. The values are similar in the two groups. In either protocol, experimenters use but a small portion of the geometrically available range of relative tolerance, the common practice being to manipulate relative target tolerance from about *W*/*D* = 0.02 or 2% to about 1/3 or 33%.

**Table 1:** Minima and maxima of relative tolerance in a sample of Fitts' law studies (*).

	Relative target tolerance *W/D*		% utilization of geometrically
Discrete protocol	MIN	MAX	available range *W*/*D* = ]0;2[
Fitts & Peterson (1964) [4]	0.010	0.333	16.1%
Kerr & Langolf (1977) [22]	0.013	0.250	11.9%
Jagacinski & Monk (1985) – Joystick [23]	0.040	0.376	16.8%
Jagacinski & Monk (1985) – Helmet [23]	0.053	0.499	22.3%
MacKenzie et al. (1987) [24]	0.011	0.333	16.1%
Andres & Hartung (1989) [25]	0.043	0.500	22.9%
Mohagheghi & Anson (2001) [26]	0.028	1.489	73.0%
*Median*	0.028	0.376	16.8%

	Relative target tolerance *W/D*		% utilization of geometrically
Reciprocal protocol	MIN	MAX	available range *W*/*D* = ]0;1[
Billon et al. (2000) [27]	0.023	0.300	27.7%
Bootsma et al. (1998) [28]	0.017	0.250	23.3%
Davis et al. (2008) [29]	0.016	1.000	98.4%
Drury (1975) [30]	0.037	0.333	29.6%
Fitts (1954) – tapping [1]	0.016	1.000	98.4%
Fitts (1954) – disc transfer [1]	0.002	0.125	12.3%
Fitts (1954) – pin transfer [1]	0.002	0.125	12.3%
Glencross & Barrett (1983) [31]	0.021	1.000	97.9%
Hoffmann & Sheik (1991) [32]	0.021	1.000	97.9%
Kerr (1973)[33]	0.014	0.167	15.3%
Kerr (1978) [34]	0.008	0.300	29.2%
Langolf et al. (1976) [35]	0.018	1.000	98.2%
MacKenzie (1991) [36]	0.016	1.000	98.4%
MacKenzie & Isokoski (2008) [37]	0.063	0.063	0.0%
Maruf et al. (1999) [38]	0.061	0.667	60.6%
*Median*	0.017	0.333	29.6%
*Median both protocols*	0.017	0.355	

(*) Note. The rightmost column presents for each study the percentage of utilization of the geometrically-available range of relative tolerance, which is not the same for the discrete and the reciprocal protocol (see Section 5.1).

**Figure 4:**
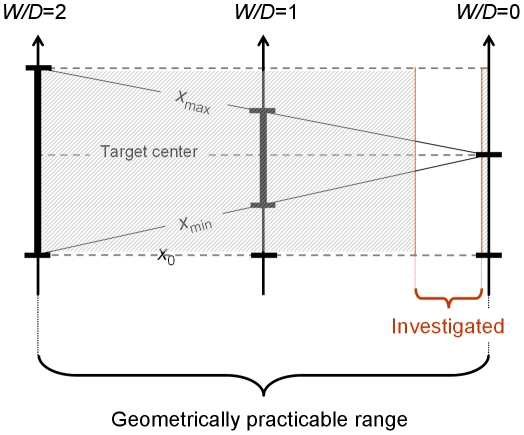
The geometrically available range of relative tolerance and the subset that is actually investigated in the Fitts paradigm of **Eq. 8**, using the discrete protocol. The *x* continuum of Figures 1–3 is now oriented vertically, with the target interval shown as a thickened segment. *W* is made to decrease from left to right for a constant value of *D*, meaning that difficulty increases from left to right. The region of relative tolerance that is not used, and presumably not usable, is hatched.

Figure 4 illustrates graphically, in the discrete-movement case, the approximate location and extent of the subset of *W/D* values that is commonly used in Fitts' law experimentation. Two facts which to our knowledge have escaped attention so far are clearly visible. First, the range covers hardly 20% of what is geometrically doable in the laboratory. Second, it is located at the extreme right of the geometrical available range, nearly touching the absolute zero of relative tolerance.

**Figure 5:**
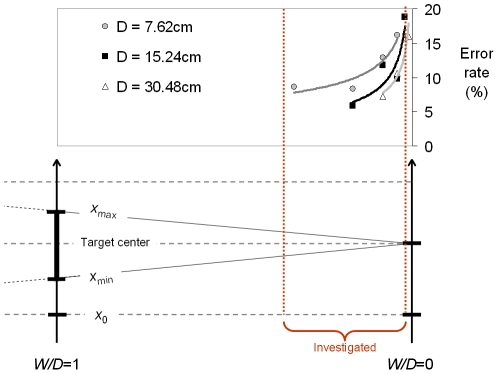
The error rate data reported by Fitts and Peterson [**4**], who manipulated relative tolerance in the range 1%≤*W/D*≤33.3%. The curves shown are power functions, whose fit is best for the three scale levels. The vertical dotted lines show the approximate location of the median maximum and median minimum of relative tolerance of Table 1. Notice that this figure and the next show only half the geometrically permissible range of *W*/*D*.

It is easy to understand in light of performance data why in Fitts' law experimentation the value of relative tolerance *W/D* can be neither much less than 2% nor much more than 1/3. Consider the 2% minimum first. Figure 5 uses the data of Fitts and Peterson [4] to illustrate the well-know fact that the frequency of target misses increases at a positively accelerated rate as task difficulty is raised. The fact is problematic since the Fitts paradigm requires by construction a constant error rate—a fixed 4%, according to MacKenzie [15]. Given the concave-up curvature of error curves, best modeled by power functions, the closer an experimenter ventures to the zero of tolerance that stands at the extreme right of Figure 5, the stronger the likely violation of instructions regarding error rates.

**Figure 6:**
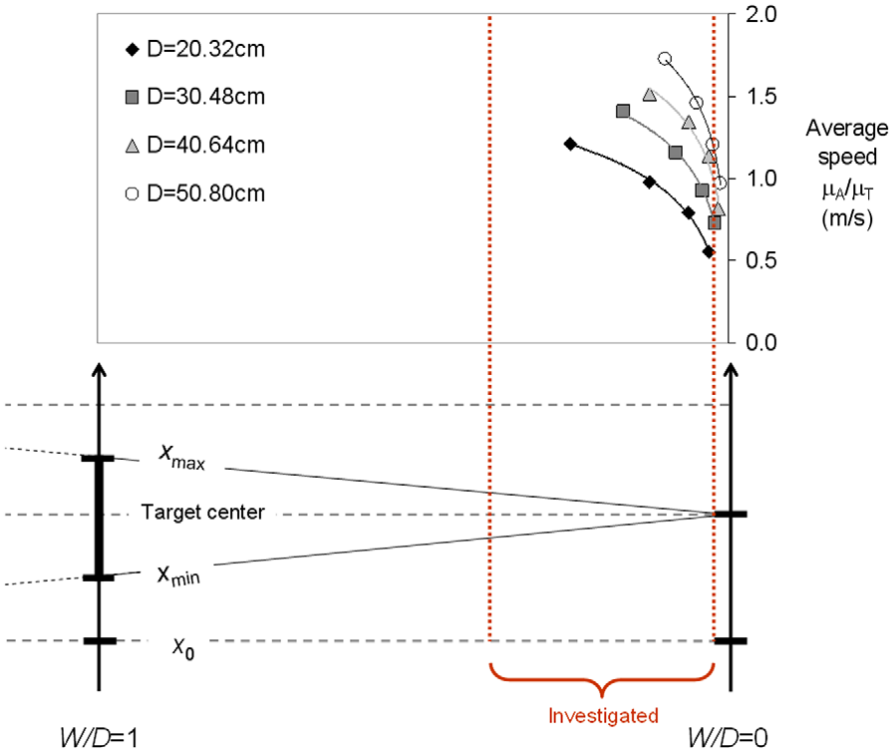
Average movement speed computed in the data of Kerr and Langolf [**22**], who manipulated relative tolerance in the range 0.013≤*W/D*≤0.25. The curves are best modeled as logarithmic functions.

Why, on the other hand, experimenters typically refrain from investigating *W/D*>1/3 can also be explained in terms of performance constraints (Figure 6). Since the Fitts paradigm requires participants to move as fast as possible for a given level of geometrical difficulty, every reduction in the accuracy demand entails an increase in the speed demand—the “easier” the task according to the *ID* criterion, the harder it actually is in terms of its mechanical energy cost [3], [39]. But the speed of an arm movement has an upper limit and therefore were task difficulty indefinitely reduced, sooner or later experimenters would face a speed-saturation effect. This effect can be anticipated in the example of Figure 6, where all curves, best modeled by logarithmic functions, exhibit highly consistent concave-down curvature. Recalling that the kinetic-energy cost of movements must increase with the square of their speed, it is not too risky to predict, by extrapolating the curves to the left, a leveling off of average movement speed somewhere beyond *W/D* = 1/3. Fitts' law is undoubtedly doomed to failure in the region situated on the left of the commonly investigated range. At a given scale level, at the point where the average speed of the movement saturates, becoming insensitive to any further increase of relative tolerance, out of necessity movement time will become insensitive to task difficulty (i.e., μ_A_ remaining about equal to *D*, a constant, the ceiling effect on μ_A_/μ_T_ implies a floor effect on μ_T_).

In sum, if the task geometry allows relative target tolerance *W/D* to be manipulated in the ]0;2[ range, in practice the range experimenters can use is actually much narrower. Due to accuracy limitations, relative target tolerance *W/D* cannot be investigated much below 2%. Due to speed limitations, *W/D* cannot be investigated much above 1/3.

## 5. The Metric of Task Difficulty

### 5.1. The Mapping of Numerical IDs onto the Task Geometry

**Figure 7:**
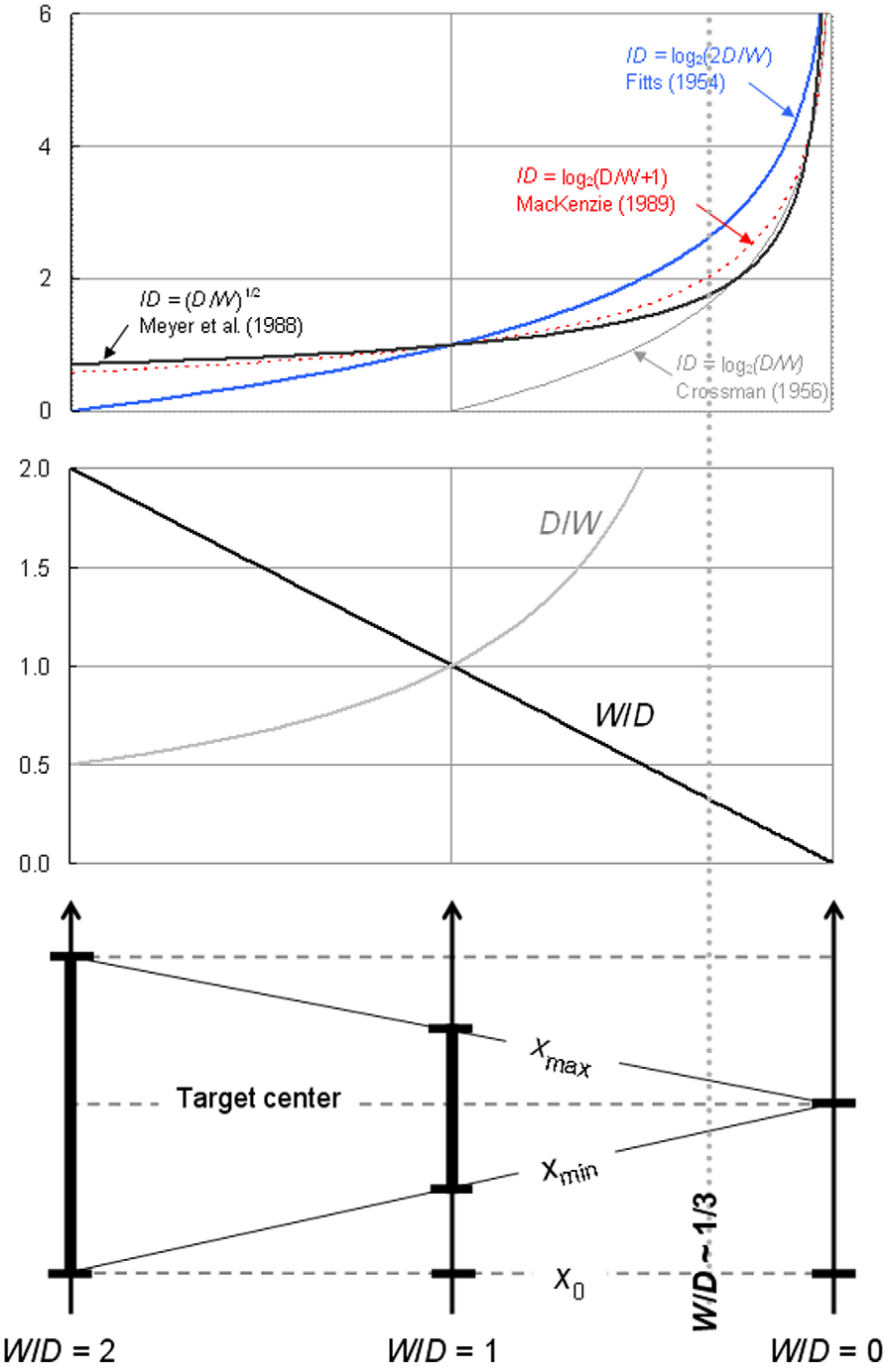
The mapping of the *ID*s of **Eqs. 3**–6 (upper panel) as well as the raw quotients of *D/W* and *W/D* (middle panel) onto the concrete geometry of a discrete aiming task (lower panel). The vertical dotted line shows the approximate location of the upper limit of relative tolerance.

Figure 7 shows how the four *ID*s of Eqs. 3–6 (upper panel) map onto the quotient from which they are computed, expressed both as relative target tolerance *W/D* and relative target distance *D/W* (middle panel), as well as onto the task geometry (lower panel). Illustrated is the whole geometrically available range of difficulty. As visible in the lower panel, the leftmost limit is *W/D* = 2, where the target begins to absorb the start point (no task, we argued, can be easier than that); the rightmost limit is *W/D* = 0, where the tolerance zeroes out (no task can be more difficult than that); right in the middle we have *D* = *W*.

We may now address the question crucial to our scale of measurement enquiry. What happens to the physical quantity of interest at the point where its numerical measure becomes zero? In the case of absolute temperature, physicists have a firm rationale for assuming that zero Kelvin corresponds to absolute freezing—at that limit atoms are assumed to no longer vibrate. Unfortunately, the picture is much less satisfying when it comes to the zero of task difficulty.

Numerically speaking, two of our four *ID*s, the Shannon *ID* (Eq. 5) and the Meyer et al. *ID* (Eq. 6), *never* zero out, as this would demand an infinite value of *W*/*D*. For example, were *W* 100 times larger than *D* in a Fitts' task, a rather absurd supposition, these two *ID*s would be 0.014 and 0.005, respectively.

The other two *ID*s do zero out, but not at the location where we would like them to. Figure 7 shows that the Fitts *ID* (Eq. 3) reaches its zero at the leftmost point where the target begins to absorb the start point (*x*_min_ = *x*_0_). This, as we said, may be viewed as a geometrical limit of practicability of the paradigm but it is emphatically not a zero of difficulty. To call this point a zero of difficulty would be to confuse the disappearance of the object of interest—the aiming task—with the cancelling out of the object's attribute that we want to measure—its difficulty. To use the absolute temperature comparison again, a piece of matter is not supposed to disappear at the point where its atoms will cease to vibrate.

Finally, the Crossman *ID* (Eq. 4) zeroes out at *D/W* = 1, obviously not a zero of difficulty.

In fact Figure 7 makes it clear that the Fitts paradigm of Eq. 8 simply does *not* allow the definition of a true, non-arbitrary zero of relative target distance *D/W*. The quotient of *D*/*W*, the basic predictor or movement time in the paradigm, therefore runs on a non-ratio (equal-interval) scale of measurement. And that conclusion extends of course to all *ID*s, as no mathematical transform of a given physical quantity can provide that quantity with a physical zero, if it lacks one.

At the rightmost limit of Figure 7 the quotient of *D/W* as well as the *ID*s that are computed from it become infinite, but at that point we do have a certain physical quantity that zeroes out, and this is target tolerance *W*. While it is impossible in the laboratory to realize *D* = 0 with *W*>0, hence *D/W* = 0, the lower panel of the figure shows that it is perfectly possible to very closely approach *W* = 0 with *D*>0, hence *W/D* = 0. Relative target tolerance *W/D* has a true physical zero, a feature that the whole variety of *ID*s proposed in the literature fail to exploit.

**Figure 8:**
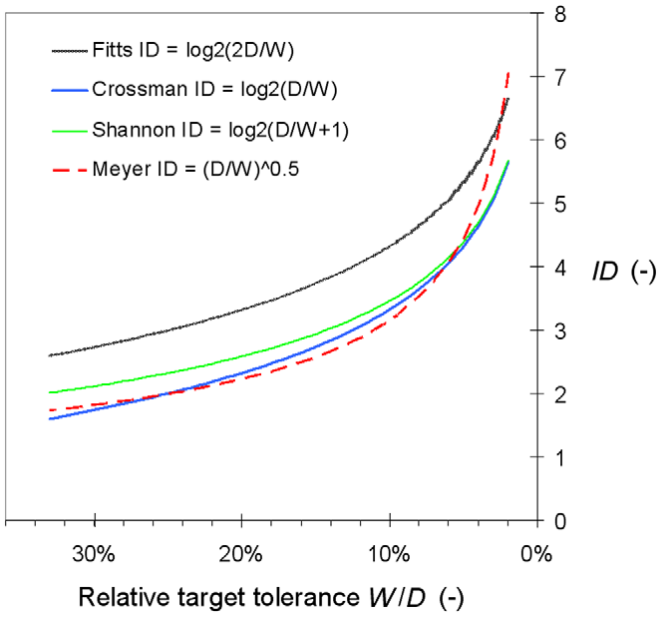
How the four *ID*s of **Eqs. 3**–6 vary in the experimentally practicable range of relative tolerance.

In Figure 7 the approximate practical minimum of difficulty *W*/*D* = 1/3 is marked by a dotted line: all the task conditions that are actually investigated in Fitts' law experimentation fall on the right of that line. Figure 8 offers a zoomed-in view of this all-important region of the continuum of relative tolerance, focusing on the 2%–33% range. Within the short range of relative tolerance that experimenters can manipulate, the four *ID*s respond rather similarly to variations of the task geometry, even though there is more curvature with the power *ID* of Meyer et al. than with the three logarithmic *ID*s. While in the preceding figure, which considered the complete range of geometrically permissible tolerances, we had distinctively different curves, now it is apparent that within the narrow interval that can be investigated all candidate *ID*s correlate rather strongly with one another. If the correlation is obviously *r* = +1 between the Crossman and the Fitts *ID*s, which vary in parallel, the lowest correlation, obtained between the Crossman or the Fitts *ID* and the Meyer *ID*, is no less than *r* = +.98. This helps understand why it is generally hard to decide, in the presence of more or less noisy data, which model fits best.

### 5.2. Arbitrariness of the y-Intercept of Fitts' Law

We have reached the conclusion that the *ID*, the predictor of Fitts' law in the standard Fitts paradigm, runs on a non-ratio (equal-interval) scale of measurement. This observation casts light on a recurrent debate of the literature about the interpretation of the *y*-intercept of Fitts' law, the coefficient *k*_1_ of Eq. 1.

Ever since Fitts, the appearance of non-zero *y*-intercepts in the plot of Fitts' law data has been a controversial topic among users of the Fitts paradigm. To explain positive intercepts, researchers have for example pointed to the time it takes to tap in place [40] or to press a mouse button [15], to dwell time [41], to an unavoidable delay in the psychomotor system [41], to uncontrollable muscle activity in the beginning or end of a movement [15], and to modeling errors such as failure to use the Shannon formulation of the *ID* or recourse to a nominal, rather than effective, measure of *W* [15], [42], or to unidentified methodological flaws [43].

But negative *y*-intercepts have also been found countless times in the literature, e.g., [4], [5], [44], [45], [46]. Of course negative intercepts in Fitts' law plots look problematic because movement time cannot conceivably be less than zero. Today the issue is still unsettled, and Soukoreff and MacKenzie (2004) [43] seem to have summarized a widespread opinion when writing that “a small intercept is a useful check that one's experimental methodology is sound” (p. 785).

One important fact that seems to have been overlooked so far is that the *y*-intercept of a linear regression—i.e., the value taken by *y* at the abscissa *x* = 0, estimated through leftward extrapolation from a necessarily finite test range—is interpretable only to the extent that *x* = 0 marks an identified physical limit. Since it is impossible in the Fitts paradigm to define a physical zero of difficulty, one has no rationale for expecting the *y*-intercept, the *k*_1_ of Eq. 1, to take any particular value. We have seen in Figure 7 that some *ID*s have numerical zeros that fall at arbitrary levels of difficulty (e.g., the Fitts and the Crossman *ID*). Others do not even have a numerical zero (e.g., the Shannon and the Meyer et al. *ID*), meaning the *y*-intercept in this case is just a graphical artifact of linear regression. In either case the *y*-intercept might perhaps serve for comparison purposes to characterize the elevation of a Fitts' law curve, given a certain *ID* range, but its empirically determined value is uninterpretable in the absolute (in order to characterize curve elevation a simple average of all μ_T_ values over one's test range presumably delivers a safer statistic than the *k*_1_ of Eq. 1 because it saves the inference of an extrapolation.). Thus the above analysis suggests that the old intercept debate of the literature has revolved about a moot point.

## 6. Grounding Task Difficulty on Relative Target Tolerance

### 6.1. Why Distinguish W/D from D/W?

Fitts' law has been formulated almost invariably in the literature as the dependency of μ_T_ upon the quotient of *D/W*. It is interesting to recall that the special relevance of that quotient had been noticed by Woodworth (1899) [47], half a century before Fitts. However, it was the *inverse* expression *W/D*—the Weber fraction, as he called it—that Woodworth called attention to. Why we carefully distinguish *W/D* from *D/W* in the present paper requires an explanation.

Mathematically speaking the distinction between the fractional expression *D/W* and its reciprocal *W/D* is idle. For example no matter whether the Fitts *ID* of Eq. 3 is noted as log(2*D*/*W*) or −log(*W*/2*D*) as these are just two different writings of the same thing. Experimental psychologists, however, do not face pure mathematics tasks. Fitts' law students do resort to mathematical modeling, but what is most important to them are physical variables. As empirical scientists, they need to care about both the abstract quantities of their formal models and the variables they concretely measure and manipulate in the laboratory, and so they need to care about the correspondence between the former and the latter.

There is indeed reason to distinguish *D/W* vs. *W/D* in the context of an experimental study of Fitts' law. Here are some arguments.

- *Semantics*. The two writings denote obviously different quantities of the real world. As already noted, the quotient of *D/W* provides a relative measure of target *distance* (i.e., *D* scaled to *W*), whereas the inverse number provides a relative measure of target *tolerance* (i.e., *W* scaled to *D*). Whether the measures be defined relatively or in the absolute, target distance is the variable experimenters use to control movement amplitude, whereas target tolerance is the variable they use to control the spread of movement endpoints.
- *Metric*. As shown in the preceding section, relative target distance *D*/*W* runs on a *non-ratio* (equal-interval) scale, with an arbitrary zero, whereas relative target tolerance *W*/*D* runs on a *ratio* scale, with a physical stop.
- *Error*. Users of the Fitts paradigm generally agree that Fitts' law amounts to a speed-accuracy trade-off, the bottom line idea being that the left- and right-hand sides of Eq. 8 convey information about performance speed and accuracy, respectively. So the expression *D*/*W* is supposed to measure accuracy in some way. But any measure of accuracy, whether absolute or relative, should involve *error* as a component, and so the right-hand side of our equations should be based on a measure of target tolerance (i.e., permitted variable error) rather than a measure of target distance (i.e., recommended amplitude). The manipulation of *D/W* is an experimental technique of forcing the quotient of μ_A_/σ_A_ to vary. Thus if Eq. 8 explicitly describes a dependency of μ_T_ upon task difficulty, a deterministic attribute of the target layout, what the equation describes ultimately is a dependency of μ_T_ upon accuracy, a stochastic attribute of the movements. Adopting the latter understanding of the law, it is more satisfactory to ground (in)accuracy on the coefficient of variation of amplitude σ_A_/μ_A_ rather than its inverse μ_A_/σ_A_, if only for a metrical reason: for any random variable *x* defined in the ]0;+∞[ interval, the coefficient of variation σ_x_/μ_x_ has a zero at the limit where, the variability vanishing out, the random variable turns deterministic; whereas the inverse quotient μ_x_/σ_x_ cannot have a zero because a positive quantity cannot have zero magnitude on average with a non-zero standard deviation.

### 6.2. Difficulty as Relative Target Intolerance

Without leaving the Fitts paradigm of Eq. 8, let us switch from relative distance to relative tolerance. This means taking the reciprocal of the fractional expression and rewriting Fitts' law as
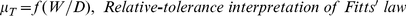
(12)where *f* now denotes some strictly *decreasing* function. We have seen in Table 1 that in the laboratory relative target tolerance *W/D* varies, roughly speaking, from 0.02 up to 1/3. The fact that experimenters cannot sensibly use target layouts such that *W/D*>1/3 or so, due to the speed saturation effect (Figure 6), means that *W* is always smaller than *D*, and hence *W/D* always smaller than 1. Thus it is convenient to express *W/D* (and of course not *D/W*), as a percentage. For example, considering the first row of Table 1, we may say that Fitts and Peterson varied relative target tolerance from 1% to 33.3%.

The target is 100% tolerant when *W* = *D*. This case being out of reach in practice, relative tolerance will always fall within the range from 0% (a true physical stop) to 100%. An interesting next step to obtain a measure of task difficulty is to convert relative target tolerance *W/D* into relative target *in*tolerance 1−*W/D*, along the lines of Meehl [6] (whose goal, in a different context, was to quantify the degree of empirical corroboration of risky numerical predictions from substantive theories). Without having to sacrifice the convenient 0–100% range of variation, we now face a clear definition of task difficulty. The higher the relative target intolerance 1−*W/D* in the Fitts paradigm, the more difficult the movement task. With this new independent variable, Fitts' law becomes a relation of the form
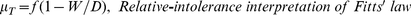
(13)where *f* now denotes some strictly increasing function. That relative target intolerance zeroes out at *W/D* = 1 is an assumption, and so our zero of relative intolerance is arbitrary. However, the upper limit of our new measure of difficulty, *total* relative intolerance (1−*W/D* = 1, or 100%), is indeed a physical stop—since *W/D* cannot be less than zero, a task with 1−*W/D*>1 is impossible.

**Figure 9:**
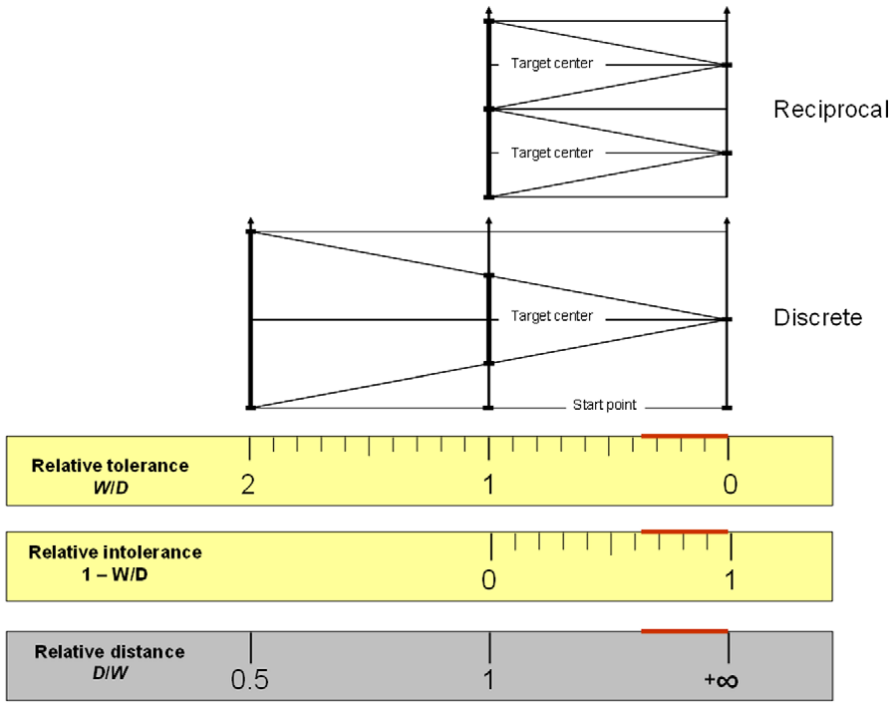
Candidate yardsticks for the measurement of movement difficulty in the Fitts paradigm. The experimentally practicable region of difficulty is marked on the edge of each graduated ruler.

Figure 9, which considers both the discrete and the reciprocal protocols, uses the metaphor of graduated rulers. Shown are our three candidate yardsticks, with their different graduation systems. Notice that the only difference between the two protocols with respect to difficulty measurement is that *W/D*>1 is geometrically possible with the discrete but not the reciprocal protocol. With the reciprocal protocol *W/D*>1 would imply target overlap, and a participant could permanently satisfy the task requirement without moving, by simply positioning the pointer somewhere in the overlap interval.

## 7. An Illustration with Fitts' (1954) Data

This section takes the example of Fitts' (1954) [1] famous stylus-tapping data to give some sense of what it means to move from the familiar *ID*-based analysis of Eq. 8 to the tolerance- and intolerance-based analysis of Eqs. 12 and 13. The data of this elegant experiment of Fitts, which he tabulated in detail, has been often used as a benchmark, e.g. in [15].

Fitts ran his famous stylus-tapping experiment twice, on two consecutive days. On Day 1 his participants used a light, 1-oz (28gr) stylus, and on Day 2 they used a heavier 1-lb (454gr) stylus. The two sets of numerical data, which Fitts tabulated in his Table 1 (p. 264), are nearly identical, but it has been a tradition in the literature to discuss the data of the light-stylus experiment. We follow this tradition here.

Fitts reported mean movement time estimates, on average over 16 participants, for each of 16 combination of *D* and *W*. Our analysis below separates the different levels of *D*, which we take as an estimate of scale [21].

**Figure 10:**
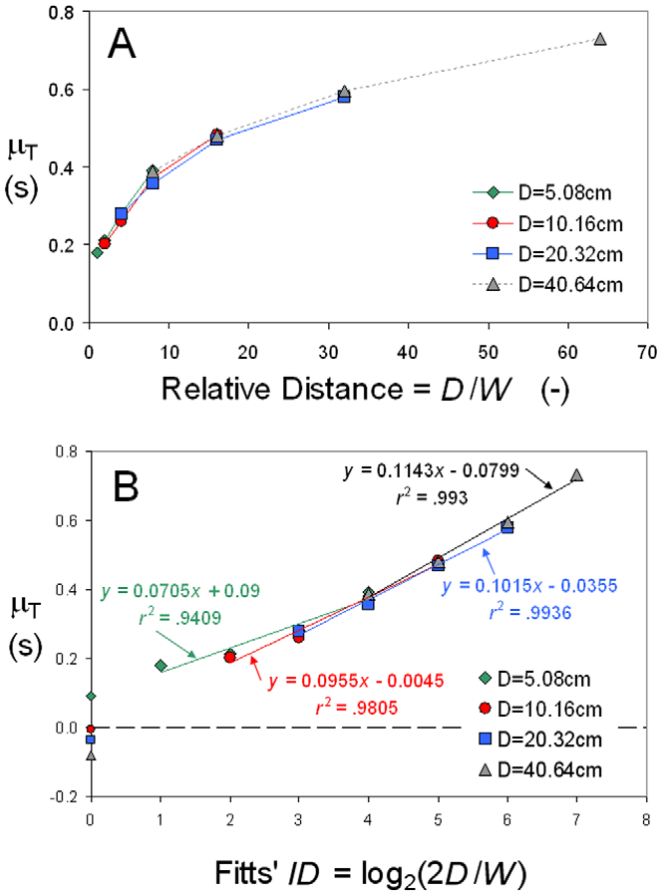
The *ID*-based description of Fitts' data, separating the four scale levels. Above: μ_T_ as a function of relative target distance specified by the raw quotient of *D/W*. Below: μ_T_ as a function of the Fitts *ID* = log_2_(2*D*/*W*). The *y*-intercept of each model equation is marked at *ID* = 0.

Figure 10 plots Fitts' data in keeping with Eq. 8, assuming that the basic predictor of μ_T_ is relative target distance *D*/*W*. Panel A uses the raw quotient of *D*/*W*, and Panel B shows that the four plots become nicely linear once the *x* axis has been transformed logarithmically. MacKenzie [15] has shown that the Shannon *ID* = log_2_(*D*/*W*+1) provides a slightly better fit of this data, and Meyer et al. [10] have noted that a power transform of *D*/*W* does yet a little better, but we must leave these observations aside to focus on the metrical issue.

The point that must be made about Figure 10B, a familiar plot of Fitts' law, is that it lacks a *physical anchor* on its *x* axis. The Fitts *ID* = log_2_(2*D*/*W*) zeroes out at *D*/*W* = ½, but we have seen that this case cannot be realized, not even approached in the laboratory, the practical minimum of *D*/*W* for the reciprocal protocol being 1 (Figure 9). The fact that three of the four *y*-intercepts turn out to be negative in Fitts' data does not matter as these estimates are uninterpretable (Section 5.2). But there is reason to bother that with this traditional *ID*-based depiction of Fitts' law one cannot respond to Soukoreff and MacKenzie's [43] above-quoted concern: the *y*-intercepts values Fitts obtained cannot help to check the soundness of his methodology.

**Figure 11:**
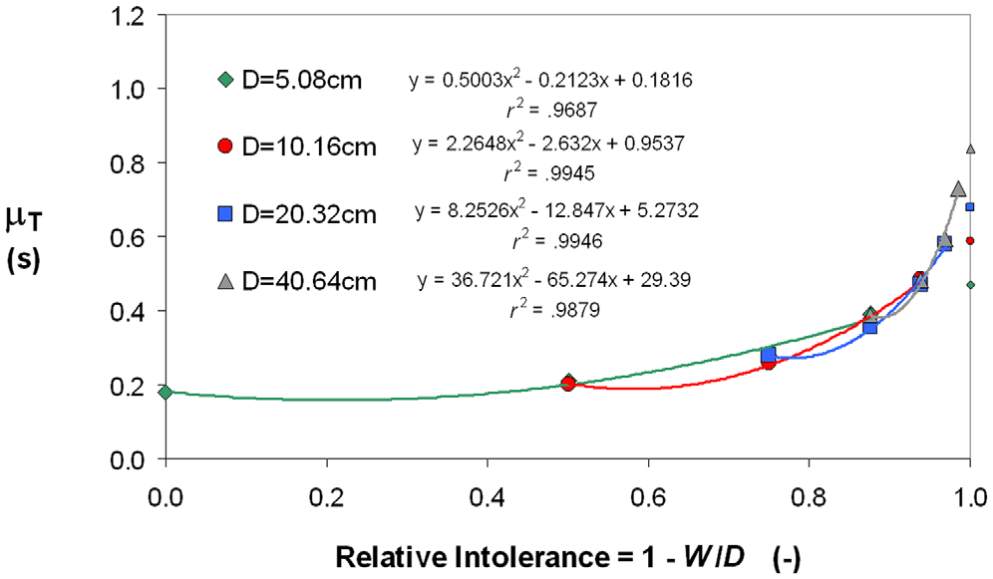
The intolerance-based description of Fitts' (1954) data. Shown at the extreme right of the plot, for each scale level, is the estimate of μ_T_ at the physical limit of 100% intolerance.

Figure 11 shows an alternative plot of Fitts' data, based on Equation 13, where the predictor of μ_T_ is relative target intolerance. Relative to Figure 10, two novelties are noteworthy.

One is that the abscissa 1−*W*/*D* = 1 corresponds to a well-defined physical maximum of intolerance (*W* = 0 with *D*>0, hence *W*/*D* = 0, hence 1−*W*/*D* = 1), meaning that rightward extrapolation to this limit delivers an interpretable estimate of curve elevation, unlike the traditional *y*-intercept of Fitts' law. Modeling the data with a polynomial *y* = *ax* ^2^+*bx*+*c*, and noticing that if *x* = 1, then *y* = *a*+*b*+*c*, it is easy to see that the sum of the three adjustable coefficients *a*+*b*+*c* provides an estimate of movement time for a *100% intolerant target*. Whereas in Figure 10 no one could explain what is meant by performance at the zero-difficulty level, what is meant in Figure 11 by performance at the 100% level of intolerance is quite clear.

The four estimates, 470 ms, 587 ms, 679 ms, and 837 ms for *D* = 5, 10, 20, and 40 cm, respectively, look quite sensible. These values suggest that scale, the paradigm's other independent variable [19], exerted a strong monotonic effect on movement time—the shorter the movement, the better the performance—indicating that this particular task of Fitts had a scale optimum located below 5 cm. However, the four estimates should be taken with caution. As usual in the Fitts paradigm, Fitts' error rates increased with the *ID*. A further concern is that in Fitts' easiest condition *W*/*D* = 1 the error rate was exactly 0%, and so one may doubt that his participants fully exploited the huge amount of tolerance made available to them in this extreme task condition. The point we want to make is that these values, unlike the *y*-intercepts of traditional Fitts' law equations, are in principle interpretable in the absolute.

The other noteworthy difference is that the extent of extrapolation required in Figure 11 to reach the (physically meaningful) 100% intolerance limit is far shorter than was required in Figure 10 to reach the (physically meaningless) zero of difficulty. This is because experimentation with the Fitts paradigm of Eq. 8, whether using the discrete or the reciprocal protocol, typically includes difficulty maxima that fall in practice in the vicinity of the zero-tolerance, or 100%-intolerance case (Table 1). A shorter extrapolation extent means a saving of inferential risk.

## 8. Discussion

### 8.1. Numbers and Physical Quantities

From our experience of discussing these issues we anticipate that some readers will be tempted to shrug off our claim that *ID*s are based on *D/W* and that because the zero of this measure is arbitrary then the Fitts paradigm has a problem. A mathematically inclined mind is likely to feel that relative target distance *D/W* and relative target tolerance *W/D* are just two different wordings of the same thing. Such a feeling, we believe, is a mistake.

**Figure 12:**
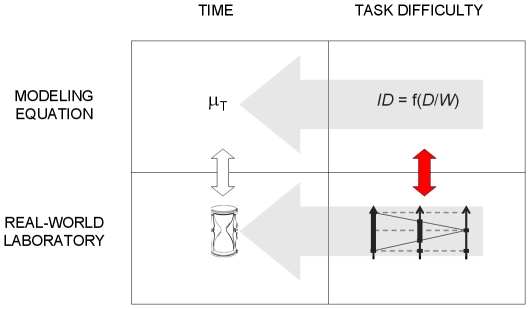
Numerical vs. physical quantities in the Fitts paradigm of **Eq. 8**.

The care to not confuse the numerical and the physical facets of difficulty is a necessity in any inquiry about measurement along the lines of S.S. Stevens [13]. It is a direct reflection of the realist postulate indispensable not just to the theory of scales of measurement [48], but to empirical science in general [49]: our equations describe the real world, which exists independently of them. As suggested in Figure 12, Fitts' law taken in the sense of Eq. 8 is a dependency of a time measure upon a task-difficulty measure (horizontal arrows). From the moment these entities and that dependency are recognized to exist in the real world, it becomes important to carefully check, as we have tried to do in the foregoing, the correspondence between the numerical quantities of our modeling equations and physically defined quantities (vertical arrows in the figure).

The fact that the numerical vs. physical distinction has remained essentially foreign to Fitts' law research seems surprising. Since Woodworth (1899) [47]—for whom Fechner's (1860) *Elements of Psychophysics* was a model to imitate—the task faced by students of simple aimed movement has been similar to that faced by psychophysicists. Both fields have endeavored to simplify their research problem (perception, movement) to the extreme, down to the point where independent variables become raw physical variables. But while psychophysics has substantially contributed to the theory of measurement [13], [48], Fitts' law research seems to have remained essentially unconcerned about measurement issues.

This is why perhaps special attention is required on the part of the reader. It may be useful to recall that it is generally unsafe to discard distinctions which, however clear-cut, have been judged unnecessary so far. As noted by Meehl [6], “one should initially disaggregate, leaving open the possibility of reaggregation if the subdivision turns out not to matter; whereas, if one begins by aggregation, one may be throwing away important information that is not recapturable” (p. 394).

Back to the example of Fitts' data, one cannot appreciate the difference between Figure 10 and Figure 11 unless one bears in mind that each of the two axes of a Fitts' law plot represents simultaneously something numerical and something physical. Focusing on the *x* axis of Figure 10B, it is undeniable, *numerically* speaking, that Fitts had a zero of difficulty in his experiment simply because log_2_(2*D*/*W*) = 0 only requires 2*D* = *W*. But the case 2*D* = *W* does not exist in the real world (Figure 9). Therefore, we argued, the zero of Fitts' *ID* is a numerical speculation.

In fact the relevance of most of the distinctions we made in the present paper depends on this fundamental numerical vs. physical distinction, to which in our view the study of Fitts' law has paid insufficient attention. If one is content with an exclusively numerical understanding of the law, then there is no need to make the following four distinctions.

1. Relative *tolerance W/D* vs. relative *distance D/W*. These are strictly equivalent quantities in mathematical formulas. We have just objected that in the physical world these designate different variables, with different scales of measurement.
2. *One quotient* vs. *two operands*. As far as standard mathematical calculations are concerned, it is convenient to leave it undetermined whether a fractional expression *n*/*d* denotes the two operands of the (doable) division of numerator *n* by denominator *d* or, alternatively, the quotient *q*, the result of the specified operation (done). In Eq. 12 the fractional notation *W*/*D* may refer to either a single number, the quotient of *W/D*, or *two* numbers, the operands *W* and *D*. What one faces here is uncertainty about *how many* real-world variables the Fitts' law formula is supposed to model [21]. From the moment one cares about how the physical meaning of the model's numerical variables, such uncertainty must be removed. This was done above with the explicit statement of Eq. 11 that Fitts' law is of the form *y* = *f*(*x*), meaning that the independent variable of a Fitts' law equation is a single quotient and that that quotient refers to a single quantity of the physical world, relative and dimensionless.
3. *Symmetrical* vs. *asymmetrical* relation. The traditional approach to Fitts' law has generally contented itself with equation models of the form *y* = *f*(*x*) where the direction of causality is left unspecified, the formula *y* = *f*(*x*) being assumed to be readily convertible into the equivalent formula *x* = *f* ^−1^(*y*). In the laboratory, however, things are less fluid. Whether Fitts' law, empirically speaking, may survive a permutation of dependent and independent variables is an open question. The Schmidt paradigm of Eq. 9 has been reporter to yield a linear relation between σ_A_ and average movement speed μ_A_/μ_T_ [2]. This empirical result is simply incompatible with the received logarithmic formulation of Fitts' law.
4. *Task variables* vs. *movement variables*. The Fitts' law literature has generally ignored the fact that target distance *D* and movement amplitude *A* are very different physical quantities. For example, Fitts [1] defined his *ID* as a function of *A*/*W* (rather than *D*/*W*), thus ignoring the fact that if tolerance *W*, a characteristic of the target layout, was under his direct control, amplitude *A* was not, being a characteristic of the behavior of his participants. Likewise, authors have not cared much about the difference between target tolerance *W* and the spread of movement endpoints σ_A_, as revealed by the frequent notation *W*_e_ to refer to “effective” or “subjective” width. With such a notation it is quite unclear whether *W*_e_ denotes a deterministic characteristic of the target layout or a stochastic characteristic of the movement. That begins to matter when one wants to know what the symbols of mathematical models stand for in reality.

### 8.2. Fitts' Law in the Face of Platt's [50] Strong Inference Challenge

It is traditional to cite Fitts' law as an exemplary achievement of experimental psychology [16], [51], [52], but one may wonder whether that tradition has done Fitts' law research much service. In science, criticisms are more useful than congratulations.

In the subtitle of a widely cited paper on what he called *strong inference*, Platt (1964) [50] pointed out that “certain systematic methods of scientific thinking may produce much more rapid progress than others”. Just recalling that research can only hope to prove the falsity of hypotheses [53] and that the most heuristic strategy is to test sets of mutually exclusive hypotheses [54], Platt argued that the spectacular progress rates of highly successful fields like molecular biology or high-energy physics is mostly due to a high degree of intolerance to everything that is conceptually or empirically inconsistent. “We measure, we define, we compute, we analyze, but we do not exclude” (p. 352). It is useful to ask to what extent Fitts' law research has been intolerant to inconsistencies.

After the pioneering study of Card, English, and Burr (1978) [55], Fitts' law has become a highly topical research subject in the field of human-computer interaction (HCI), quite probably because target acquisition, since the advent of the mouse in the early nineteen eighties, has been the fundamental building block of graphical user interfaces [20]. HCI researchers and basic experimental psychologists have used the same methodology, resorting to the Fitts paradigm of Eq. 8. Yet the two communities have persistently disagreed on the mathematical formulation of Fitts' law. While the Shannon version of Fitts' law (Eq. 5), in the wake of MacKenzie (1992) [15], has been unanimously accepted in HCI, psychologists have continued to hold tight to the Fitts version of the law (Eq. 3). But pluralism seems undesirable in empirical science. To paraphrase Platt [50], “a failure to agree for 30 years is public advertisement of a failure to disprove” (p. 351).

We have seen that over the usual range of task difficulties the Fitts and the Shannon *ID*s correlate very strongly with each other (*r* = +.98, see Figure 8). Arguing that the Fitts vs. Shannon *ID* issue is of great theoretical import, Soukoreff and MacKenzie [56] conceded that it is quite hard to settle empirically. In fact one may wonder whether the issue is empirically decidable at all within the conventional paradigm, if only because no information can be drawn from the elevation of a Fitts' law curve, for lack of a physical zero on the *x* axis.

The Fitts' law literature has also tolerated for two decades an overt inconsistency between its favorite substantive theoretical explanation and its favorite mathematical description of the law. In both the HCI and the experimental psychology communities the most widely received explanation of Fitts' law has been the stochastic optimized sub-movement theory of Meyer et al. [10]. The Meyer et al. [10] approach and the Fitts-MacKenzie information-theoretic approach are not mutually incompatible, as the former construes the movement as a stochastic process whose detailed mechanisms unfold in time whereas the latter treats that complexity as a black box, trying to predict performance directly from the properties of target layouts [54]. But the problem is that Meyer et al. claimed that their theory predicts a power relation while both communities have never ceased to assume that Fitts' law is logarithmic.

Again, there is no guarantee that the logarithmic vs. power issue is empirically decidable with the traditional Fitts paradigm. The power *ID* of Eq. 6, which strongly correlates with the logarithmic *ID*s of Eq. 3 or 5, begins to behave in a distinctive way in the region of lowest tolerance, for *W*/*D*<10% or so (Figure 8) and so the highest levels of difficulty would seem crucial. But unfortunately this is the region of tolerance where participants are most unable to comply with the accuracy instructions transmitted to them via the target layout (Figure 5).

The adjustment for errors obviously cannot help, quite the contrary, because with that adjustment the range of difficulty will inevitably shrink: in the presence of inflated error rates for higher *ID*s, this adjustment will result in the *leftward* shift of abscissas, meaning the Fitts' law plot will lose its data points in the crucial upper region of difficulty. The fact is, nearly a quarter century after Meyer et al.'s (1988) [10] proposal it is still unclear whether Fitts' law, as assessed in the conventional Fitts paradigm, is a power or a logarithmic law.

We believe that it is not Fitts' law in and of itself that suffers a measurement problem, but rather the particular version of the law that rests on the Fitts paradigm of Eq. 8, which construes the law as a dependency of movement time upon task difficulty. Obviously the Schmidt paradigm of Eq. 9 is immune to the criticism. Its dependent variable, variable error σ_A_, and its independent variable, average movement speed *A*/*T*, both have a physical zero. Immune too is the tradeoff paradigm of Eq. 10, which states the law as a relation between mean movement time μ_T_ and relative variable error σ_A_/μ_A_, two stochastic quantities that have a zero in the physical world.

Thus our analysis raises doubts about the strength of the traditional Fitts paradigm for studying Fitts' law. The weakness which we have focused on above is the failure to provide difficulty with a physically zero, meaning under-constraining empirical data. As a tentative solution to this problem, we proposed an alternative definition of difficulty based on relative target intolerance 1−*W*/*D*, rather than relative target distance *D*/*W*. But this may not suffice, as the paradigm suffers from another constitutional weakness. Not only does the Fitts paradigm measure its object in a questionable manner, one may doubt it measures the right thing.

To progress in the understanding of the tradeoff of movement speed and accuracy, one should ask whether task difficulty is the right quantity to consider. The Fitts paradigm heavily relies on the technical assumption that task difficulty, well captured by the quotient of *W*/*D*, controls performance accuracy, well captured by the coefficient of variation σ_A_/μ_A_. But that assumption has been repeatedly challenged in the literature. It is notorious that target-layout manipulations have generally provided experimenters with mediocre control over movement accuracy, and hence movement speed. The gradual inflation of target misses as relative tolerance is reduced means that *W/D* overestimates σ_A_/μ_A_ more and more. In the opposite direction, near the supposedly easy end of the range, we have the problem that error rate is often exactly 0%, as for example in Fitts (1954) data, in which case *W*/*D* probably *under*estimates σ_A_/μ_A_. Obviously, were experimenters very strict about participant compliance with their error instructions, the range of relative tolerance in the Fitts paradigm would be just a small fraction of that which has been usually investigated.

For decades the response to this concern has been the so-called adjustment for errors procedure [14], [15], [42]. All nominal values of *ID* of the experiment are recalculated in light of performance data so as to obtain an *effective ID* based on the statistics μ_A_ and σ_A_ in lieu of the geometrical measures *D* and *W*. However sensible, the procedure complicates the Fitts paradigm with auxiliary assumptions and in a sense undermines it. For example, to compute effective widths from observed distributions of endpoints, MacKenzie [15] suggested a method based on information theoretic concepts, but that method presupposes Gaussian distributions. Unfortunately the Fitts' law literature reports consistent departures from normality in distributions of movement endpoints, with both the kurtosis and the skewness varying with the *ID* [57], [58]. Thus the adjustment for errors, meant to correct a shortcoming of the paradigm, is liable to introduce extra noise in the data.

Platt [50] pointed out that the widespread habit of attaching proper names to theories and methods tends to slow down scientific progress because disagreement becomes a conflict between persons, rather than ideas. The paradigm we are discussing is a legacy of Paul M. Fitts, a prestigious figure of psychology. But an experimental tool whose efficiency has proved doubtful should not be salvaged at any cost.

In our view the traditional Fitts paradigm is quite valuable in applied research, notably HCI and ergonomics, where experimenters often need to predict pointing performance from the geometry of interfaces. When the researcher's problem is to optimize target acquisition with various input devices and various interaction techniques, the quantitative characterization of the difficulty of a target arrangement is certainly a necessity, justifying the Fitts paradigm. In such contexts we feel the measurement of relative intolerance 1−*W*/*D* has promise.

But we believe that the basic research front should investigate other directions. Rather than resort to post-hoc corrections to offset errors resulting from a shortcoming of the paradigm, it seems sensible to look for other experimental approaches where the mismatch between what experimenters prescribe to their participants and what they obtain from them does not arise in the first place. The important step is to get rid of the hardly tenable assumption that it is possible to control the accuracy of movement by means of task geometry manipulations.

This step is taken in the trade-off paradigm of Eq. 10, which uses a two-line instead of a three-line display, thus prescribing movement scale μ_A_ but not relative variable error σ_A_/μ_A_. This feature exploits the fact that if *W* is a notoriously unreliable controller of σ_A_, *D* controls μ_A_ fairly reliably [14], [15], [42], as reported again in [3]. Scale being specified, the participants' task is to concurrently minimize two quantities, movement time μ_T_ and relative variable error σ_A_/μ_A_. The crucial manipulation, orthogonal to the manipulation of scale, then consists of verbal instructions that encourage the participants to modulate their speed/accuracy imbalance over as large a range as possible, from attempts to move as fast as possible to attempts to move as accurately as possible. Notice that this instructional variable, which does not participate in the statement of Fitts' law, is just ordinal.

Exploiting a simple limited-resource model consonant with Norman and Bobrow's [59], [60] and using the trade-off paradigm, Guiard et al. [3] obtained data suggestive of a square-root relationship, with individual *r* ^2^ values ranging from .89 to .97 despite the flexibility reduction entailed by the sacrifice of one adjustable coefficient [61]:
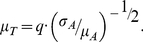
(14)


Rewriting Eq. 14 as a constant product
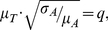
(15)the authors argued that *q*, the single free coefficient of Eq. 14, can be taken as an estimate of the amount of resources invested by the participant in the movement task. As the emphasis is gradually shifted from maximal speed to maximal accuracy, the quantity *q* is essentially conserved; what varies systematically is the quotient of μ_T_/(σ_A_/μ_A_)^½^, which they argued is interpretable as a quantitative estimate of the strategic imbalance.

One of the reasons why this candidate version of Fitts' law seems worthy of consideration is that it is based on a direct estimation of the speed and accuracy of simple aimed movements. Task difficulty having disappeared from the scene, the scale of measurement and the validity problems which, we suggested, jeopardize the traditional approach to Fitts' law are avoided.
